# “How Is My Child’s Asthma?” Digital Phenotype and Actionable Insights for Pediatric Asthma

**DOI:** 10.2196/11988

**Published:** 2018-11-30

**Authors:** Utkarshani Jaimini, Krishnaprasad Thirunarayan, Maninder Kalra, Revathy Venkataraman, Dipesh Kadariya, Amit Sheth

**Affiliations:** 1 Ohio Center of Excellence in Knowledge-enabled Computing (Kno.e.sis) Department of Computer Sciene Wright State University Dayton, OH United States; 2 Dayton Children Hospital Dayton, OH United States

**Keywords:** digital phenotype, actionable insights, asthma control level, asthma control test, digital phenotype score, controller compliance score, mobile health

## Abstract

**Background:**

In the traditional asthma management protocol, a child meets with a clinician infrequently, once in 3 to 6 months, and is assessed using the Asthma Control Test questionnaire. This information is inadequate for timely determination of asthma control, compliance, precise diagnosis of the cause, and assessing the effectiveness of the treatment plan. The continuous monitoring and improved tracking of the child’s symptoms, activities, sleep, and treatment adherence can allow precise determination of asthma triggers and a reliable assessment of medication compliance and effectiveness. Digital phenotyping refers to moment-by-moment quantification of the individual-level human phenotype in situ using data from personal digital devices, in particular, mobile phones. The kHealth kit consists of a mobile app, provided on an Android tablet, that asks timely and contextually relevant questions related to asthma symptoms, medication intake, reduced activity because of symptoms, and nighttime awakenings; a Fitbit to monitor activity and sleep; a Microlife Peak Flow Meter to monitor the peak expiratory flow and forced exhaled volume in 1 second; and a Foobot to monitor indoor air quality. The kHealth cloud stores personal health data and environmental data collected using Web services. The kHealth Dashboard interactively visualizes the collected data.

**Objective:**

The objective of this study was to discuss the usability and feasibility of collecting clinically relevant data to help clinicians diagnose or intervene in a child’s care plan by using the kHealth system for continuous and comprehensive monitoring of child’s symptoms, activity, sleep pattern, environmental triggers, and compliance. The kHealth system helps in deriving actionable insights to help manage asthma at both the personal and cohort levels. The Digital Phenotype Score and Controller Compliance Score introduced in the study are the basis of ongoing work on addressing personalized asthma care and answer questions such as, “How can I help my child better adhere to care instructions and reduce future exacerbation?”

**Methods:**

The Digital Phenotype Score and Controller Compliance Score summarize the child’s condition from the data collected using the kHealth kit to provide actionable insights. The Digital Phenotype Score formalizes the asthma control level using data about symptoms, rescue medication usage, activity level, and sleep pattern. The Compliance Score captures how well the child is complying with the treatment protocol. We monitored and analyzed data for 95 children, each recruited for a 1- or 3-month-long study. The Asthma Control Test scores obtained from the medical records of 57 children were used to validate the asthma control levels calculated using the Digital Phenotype Scores.

**Results:**

At the cohort level, we found asthma was very poorly controlled in 37% (30/82) of the children, not well controlled in 26% (21/82), and well controlled in 38% (31/82). Among the very poorly controlled children (n=30), we found 30% (9/30) were highly compliant toward their controller medication intake—suggesting a re-evaluation for change in medication or dosage—whereas 50% (15/30) were poorly compliant and candidates for a more timely intervention to improve compliance to mitigate their situation. We observed a negative Kendall Tau correlation between Asthma Control Test scores and Digital Phenotype Score as −0.509 (*P*<.01).

**Conclusions:**

kHealth kit is suitable for the collection of clinically relevant information from pediatric patients. Furthermore, Digital Phenotype Score and Controller Compliance Score, computed based on the continuous digital monitoring, provide the clinician with timely and detailed evidence of a child’s asthma-related condition when compared with the Asthma Control Test scores taken infrequently during clinic visits.

## Introduction

Asthma is the second most common chronic disease in the pediatric population. It incurred a total annual direct health care cost of more than US $80 billion dollars burdening the US economy [1]. As of 2016, more than 26 million Americans have been diagnosed with asthma, of which 8.3% are children [2]. Asthma is a multifactorial disease, manifesting many symptoms, which reduce the quality of life. The onset of asthma and the factors that affect its severity and control level vary. Asthma can affect a child’s physical and mental well-being. It can lead to reduced activity, missed school days, difficulty in concentrating, feeling isolated from peers, and in extreme cases, emergency room visits, ultimately impacting long-term academic, economic, and physical growth [3]. As such, parents want to know how well controlled their child’s asthma is on a regular basis, so that they, with the help of the clinician, can better plan and manage their child’s care protocol.

Asthma patients are usually given an asthma action plan to deal with asthma symptoms and a detailed education about triggers and the importance of compliance. It would be helpful for the parent and the clinician to receive information about the child’s adherence to care protocol and exposure to factors that can exacerbate the child’s asthma in a timely manner. We built the *kHealth system* consisting of *kHealth kit, kHealth cloud,* and *kHealth Dashboard*; adapted it for continuous and comprehensive monitoring of a child’s symptoms, activity, sleep pattern, potential environmental triggers, and medication compliance; and derived insights to help manage asthma.

The *kHealth kit* consists of a mobile app, provided on an Android tablet, that asks timely and contextually relevant questions related to asthma symptoms, medication intake, reduced activity because of symptoms, and nighttime awakenings; a Fitbit to monitor activity and sleep; a Microlife Peak Flow Meter to monitor the peak expiratory flow and forced exhaled volume in 1 second; and a Foobot to monitor indoor air quality. The *kHealth cloud* stores personal health data and environmental data collected using Web services. The *kHealth Dashboard* interactively visualizes the collected data. In the contemporary clinical protocol, the most relevant information will likely come from an Asthma Control Test (ACT) that is administered during the child’s clinic appointment. The ACT score is a recapitulation of the past 4 weeks of the child’s health condition; there is considerable concern about its ability to provide a clear picture of the child’s current health condition [4,5]. Our kHealth system seeks to remedy this limitation by embodying the digital phenotype.

The term digital phenotype refers to *moment-by-moment quantification of the individual-level human phenotype in situ using data from personal digital devices, in particular, mobile phones* [6]. The data can be collected either by the active involvement of a user, referred to as *active sensing,* or automatically and nonintrusively using sensors, referred to as *passive sensing* [7]. *Digital phenotype* in our study is the data collected through active and passive sensing of a child, resulting from the interaction of the child with the environment and medication using the kHealth kit. Previous studies have reported inaccuracies in a child’s self-reporting (in surveys, questionnaire, etc) at the clinic [8,9]. Our kHealth system overcomes this limitation by allowing 24/7 continuous and objective monitoring of a child. Digital phenotype obtained can help the clinician better diagnose, monitor, and manage asthma [6,10].

In this study, we focus on the cohort-level analysis of the data collected from 95 asthmatic children using the kHealth app. To abstract the digital phenotype in a form that is both accessible and can serve as a proxy for the current practice, we define a Digital Phenotype Score (DPS) and Controller Compliance Score (CCS). DPS and CCS are based on the comprehensive physical, environmental, activity, symptomatic, and medication intake data collected from the kHealth system and form the basis for actionable insights. For instance, unlike an ACT score, DPS and CCS together can help the parent and the clinician to intervene, improve the care protocol, change the medication dosage, and take preventative measures as needed—such as avoiding the outdoors and the use of an air filter or dehumidifier in a timely manner.

## Methods

### Study Recruitment

**Figure 1:**
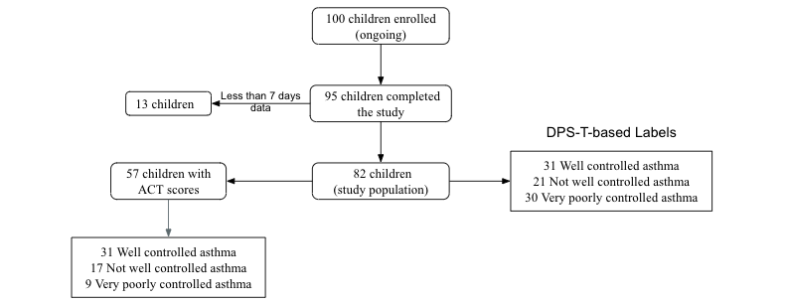
Study recruitment detail. ACT: Asthma Control Test; DPS-T: Digital Phenotype Score calculated using Total Symptom Score.

Study participants were recruited from children (within the age group of 5-17 years) diagnosed with asthma by an asthma specialist at Dayton Children’s Hospital (DCH). Study coordinator approached the parents of asthma patients seeking treatment at DCH. The parent, along with the child, consented to participate in our study. The parent provides the consent, and the child provides an assent by signing a consent form to take part in our study and giving permission to obtain their medical details from the electronic medical records (EMRs). The recruitment for the study is random, with the only prerequisite of suffering from asthma. The types of asthma, such as nonpersistent, persistent, exercise-induced, and nonexercise-induced, are not taken into account. A total of 100 study participants, along with a parent or guardian, consented to the ongoing study during December 2016 until July 2018. A total of 95 children of the 100 completed the study, with 5 children still collecting data at the time writing of this study. The child and the parent were given an option to participate either in a 1-month (n=70) or 3-month (n=25) study. The participants who completed the 1- and 3-month study were given an incentive of $50 and $100 gift cards, respectively. Figure 1 describes the study recruitment. Except for the duration of participation, all other aspects of the participants were identical. Each consenting participant was given a demonstration of all the components of the kHealth kit with access to a user guide and tutorial video on the tablet to make it accessible to both the child and the parent. In case of any trouble encountered during the study participation, the contact information of the nurse practitioner was provided to the parent. In case of technical difficulties, contact information of the kHealth team was provided in the user guide to telephonically resolve the issue while keeping the identity of the participant anonymized.

### Study Design

This is an observational longitudinal study involving collaboration among researchers from Kno.e.sis—an Ohio Center of Excellence for BioHealth Innovations at Wright State University and DCH, the latter consisting of a clinician and a nurse coordinator. The study was approved by the DCH institutional review board (IRB). The study uses readily available sensors and widely used technologies. The study comprises 30 kits to allow parallel participations of up to 30 children.

### Study Kit

#### kHealth

The kHealth system comprises a kHealth kit, kHealth cloud, and kHealth Dashboard.

##### kHealth Kit

**Figure 2:**
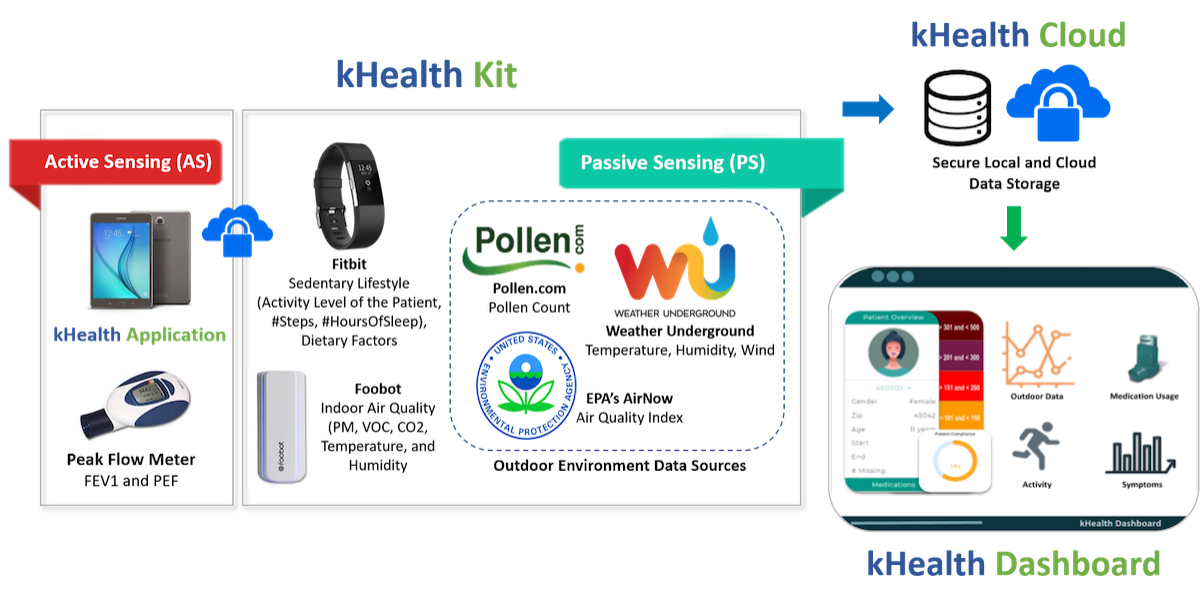
kHealth System.

A kHealth kit, as shown in Figure 2 (more information can be found in Multimedia Appendix 1), consists of an Android tablet hosting kHealth app and a set of sensors (Fitbit, Foobot, and Microlife Peak Flow Meter), which are used by the child. Sections below provide a description of all the sensors and the type of data collected from them. The data are collected using the active and passive sensing techniques. Active sensing refers to the data collection where a child (or a parent) has to actively interact with the technology (eg, answering questions after invoking a mobile app). Passive sensing refers to the data collected without active human interaction with the technology (eg, Foobot [11] sensor in a room automatically collects the indoor air quality data).

**Table 1:** kHealth app questionnaire.

kHealth app questions	Multiple-choice options
Are you currently experiencing any of the asthma-related symptoms below?	Cough, wheeze, chest tightness, hard and fast breathing, nose opens wide, cannot talk in full sentences, others
How many times did you take albuterol inhaler today because of asthma symptoms?	1, 2, 3, 4, 5, 6+
Have you had a wheeze, chest tightness, or asthma-related cough today?	Yes, no
How much did asthma or asthma symptoms limit your activity today?	None, a little, most of the day, at least half of the day
Did you take albuterol last night because of a cough or wheeze?	Yes, no
Did you wake up with a cough or wheeze last night?	Yes, no
Rescue medication question. For example, did you take albuterol today?	Yes, no
Controller medication question. For example, did you take Dulera today?	Yes, no

##### kHealth App

The kHealth app was built on the widely used Android platform [12]. The app was designed in consultation with the clinician, tested with trial patients, and iteratively refined before reaching the current version used in this study [13]. The app asks questions similar to the ACT. The app captures symptoms, medication intake, and activity limitation because of asthma symptoms and nighttime awakenings using a questionnaire that the child is expected to answer twice a day. The app was customized for every child, such as the medication (rescue and controller) information for every child was taken from the EMRs, and the medication intake questions were asked for the prescribed medication only. Table 1 below shows the kHealth app questionnaire.

##### kHealth Cloud

The data collected from the kHealth kit are synced in real time with Firebase, a Google cloud storage. Firebase provides active data listening for the client-side, which offers data persistence over a network failure and re-syncs to the cloud when the network is restored. The kHealth app uses SQLite as the primary data storage and Firebase as the secondary data storage. Data from Firebase are available to Kno.e.sis researchers and clinicians for real-time analysis. For securing remote data, Firebase provides a set of real-time database rules and user authentication that allow data access control on a per-user basis. Moreover, it is built on the Google Cloud Platform, sharing the same level of data security [14].

##### kHealth Dashboard

**Figure 3:**
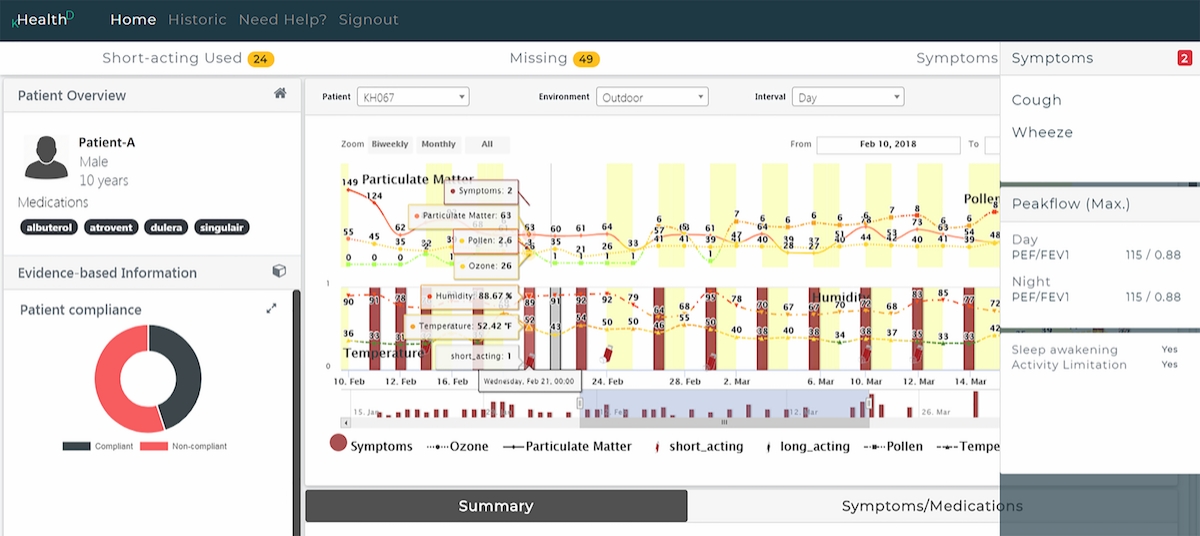
kHealth Dashboard Visualizing the Data.

kHealth Dashboard as shown in Figure 3 is a cloud-based platform that integrates and visualizes multimodal data from the kHealth kit [15] (A demo video of the kHealth Dashboard can be found by accessing the link provided in Multimedia Appendix 1). It provides an alternative to the traditional episodic clinician-centric health care by supporting real-time monitoring of a child’s health condition [16]. It is a step toward exploring issues such as the following: Can we predict asthma attacks based on the data collected from the child? Can we understand the causal relationship between symptoms and possible triggers or factors responsible for them? kHealth Dashboard allows us to visually explore the correlations between the child’s recorded readings about their condition and environmental data. It also gathers empirical evidence to analyze and monitor disease progression as well as help manage asthma.

### Study Variables

#### Symptom Score

Symptom score (SS) is the measure of the symptoms (cough, wheeze, chest tightness, nose open wide, and hard and fast breathing) experienced by the child during the study period. The study period is defined as the number of days the child took a kHealth app reading. The clinician can use the SS measure to get real-time insight into the child’s condition, such as changes in symptoms because of weather or outdoor condition. We used 2 different metrics to calculate a child’s SS based on (1) the total number of symptoms experienced by the child during the study period (the total number of symptoms experienced is the same as the total number of symptom questions answered in affirmative per day) and (2) the total number of days the child experienced some symptom during the study period. We define Total Symptom Score (TSS) as the average number of symptoms experienced by the child during the study period. We define Partial Symptom Score (PSS) as the fraction of the number of days the child experienced symptom during the study period.

Total Symptom Score (TSS) = Number of symptoms experienced by the child/Study period

Partial Symptom Score (PSS) = Number of days the children experienced symptoms/Study period

#### Rescue Score

The kHealth app collects data on the intake of the rescue medication (short-acting bronchodilators) by asking questions such as Did you take albuterol today? We define Rescue Score (RS) as the number of times the child took the rescue medication during the study period. Usually, children take rescue medication to mitigate or prevent the symptoms.

Rescue Score (RS)= Number of rescue medication intake by the child during the study period/Study period

#### Controller Compliance Score

The kHealth app asks questions (eg, Did you take DULERA today?) about the intake of the controller medication (long-term control medication). Each child is prescribed a controller medication, which they are supposed to take at least once a day. Thus, the CCS is defined as the fraction of the number of days the child took the controller medication during the study period.

Controller Compliance Score (CSS) = Number of controller medication intake by the child during the study period/Study period

**Figure 4:**
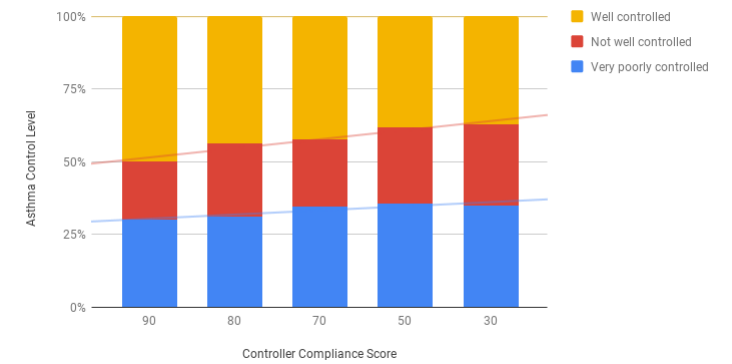
The variation of the percentage of children in the different asthma control level with the Controller Compliance Score.

The percentage of children in the well-controlled category increases with the increasing CCS, as shown in Figure 4. The more compliant children are toward their medication, the better controlled is their asthma. Table 2 describes the controller medication compliance threshold. We chose a threshold of 70%. For example, if the child took her/his medication at least 70% of the prescribed duration during the study period (for 1 month [30 days] minimum 21 days and for 3 months [90 days] minimum 63 days), then the corresponding CCS≥0.70 and is the child is classified as *highly compliant*.

**Table 2:** Controller Compliance Score thresholds.

Compliance	Controller Compliance Score (CCS)
Highly compliant	CCS≥0.70
Well compliant	0.30≤CCS<0.70
Poorly compliant	CCS<0.30

#### Activity Score

The Activity Score (AcS) is the number of days the child had restricted activity during the study period. The kHealth app asks a multiple-choice question: *How much did asthma symptoms limit your activity today?* with 4 options: none, a little, half of the day, and most of the day. The options are given a weight on a scale of 0-3, respectively.

Activity Score (AcS) = Summing over the days in the study period (activity restriction presence option weight)/Study period

#### Awakening Score

The Awakening Score (AwS) is the number of nights the child woke up because of asthma symptoms during the study period. The kHealth app asks a multiple-choice question: *Did you wake up with wheeze, cough, or any asthma-related symptoms?*

Awakening Score (AwS) = Number of nights the child woke up with asthma symptoms during the study period/Study period

#### Asthma Control Test Score

ACT is a short multiple-choice questionnaire consisting of 5 questions: about a child’s symptoms, nighttime awakenings because of symptoms, number of days the child missed school because of symptoms, intake of rescue medication, and the child’s rating about their asthma control in the past 4 weeks. Each question is given a score of 1-5, with 1 being the worst and 5 being the best. The ACT score is calculated by adding the scores for each question. Its score helps the clinician classify the child’s asthma as well controlled, not well controlled, or very poorly controlled [17]. The ACT score based asthma control level label varies with the age group. For children less than 12 years, if the ACT score is ≤12, the child’s asthma is *very poorly controlled*. If the ACT score is ≥13 and ≤19, the asthma is *not well controlled*, and if the ACT score is ≥20, the asthma is *well controlled*. For the children aged ≥12 years, if the ACT score is ≤15, the child’s asthma is *very poorly controlled*. If the ACT score is ≥16 and ≤19, the asthma is *not well controlled*, and if the ACT score is ≥20, the asthma is *well controlled*.

#### Digital Phenotype Score

Digital Phenotype Score Total (DPS-T) = Total Symptom Score + Rescue Score + Activity Score + Awakening Score

Digital Phenotype Score Partial (DPS-P)= Partial Symptom Score + Rescue Score + Activity Score +Awakening Score

The DPSs (DPS-T and DPS-P, discussed below) capture an aggregate of each of the contributing digital phenotypes that impact the child’s health. This is in the same vein as ACT, which tries to quantify the child’s asthma control based on responses to the 5 asthma condition–related questions and Patient Health Questionnaire-9 (PHQ-9), which tries to quantify patients’ depression severity based on their responses to the 9 mental health condition–related questions [18,19]. The DPSs are a digital measure of the *quality of life* of the child appropriate for the kHealth context and can be used as a succinct proxy to determine the control level. We try to capture the intuition that the higher the manifestation of asthma signs, the worse the control. The more occurrences of symptoms, increased intake of rescue medication, restriction of daily activities and nighttime awakenings, and the worse is the child feeling, the poorer is the asthma control. We propose to define the scores to reflect the cumulative effect of various negative factors impacting a child’s health and its manifestation, as measured by symptoms such as cough and wheeze, reduced physical activity, nighttime awakenings, and intake of rescue medication, either to remedy symptoms or to prevent them. Specifically, we consider 2 different alternatives for computing the score each with its own pros and cons: (1) DPS-T to capture the total number of asthma episodes by summing the TSS, RS, AcS, and AwS, which we regard as a fine-grained reflection of the child’s suffering, and (2) DPS-P that substitutes the TSS with a PSS in DPS-T (ie, DPS-P=DPS-T − TSS + PSS) to better match the ACT scoring approach to determine the control level, for fair comparison and validation with respect to the current practice. Similar to the ACT scoring, our approximation does not have differential weighing of symptoms (such as cough < wheeze < chest tightness). Although this may be adequate to obtain a coarse-grained classification for control level, it may not truly reflect the relative *quality of life* enjoyed by the child faithfully. For instance, a child having 10 coughs each day for 2 days may be better off than 10 coughs over 3 days. DPS-T better captures this intuition than DPS-P. However, ACT scores, DPS-T, and DPS-P do not satisfactorily capture the fact that someone having wheezing, shortness of breath, and chest tightness on a day may be significantly worse off than someone coughing several times a day. In fact, the former may lead to an asthma attack, even requiring an emergency hospital visit compared with the latter. In this study, we evaluate DPS-T and DPS-P as an approximate measure of the asthma-related health condition of the child.

The National Heart Lung Blood Institute (NHLBI) provides a guideline to classify the child’s asthma control level as *very poorly controlled*, *not well controlled*, and *well controlled*. We used the NHLBI guidelines and the data collected from the kHealth app to determine the child's control level by developing a comparable threshold for DPS-P and DPS-T.

Using the NHLBI guidelines given in Table 3 [20], we have developed thresholds for DPS (DPS-P and DPS-T) as shown in Table 4 (eg, if the DPS≥1, then the child’s asthma is very poorly controlled). The thresholds for the 3 control levels have been chosen to make the DPS-P and ACT scores comparable when they are available over the same period. The control levels obtained based on DPS-T using the same thresholds seem to be relatively pessimistic, but in reality, they have the potential to capture the child’s reaction to asthma triggers over the day at a fine-grained level.

**Table 3:** Modified National Heart Lung Blood Institute asthma control level classification guide (given by National Heart Lung Blood Institute).

Component of control		Age in years	Level of asthma control		
			Well controlled	Not well controlled	Very poorly controlled
**Impairment**					
	Symptoms	0-11	≤2 days/week but ≤1 time a day	>2 days/week or multiple times on ≤2 days/week	Throughout the day
		≥12	≤2 days/week	>2 days/week	Throughout the day
	Nighttime awakenings	0-4	≤1 time a month	>1 time a month	>1 time a week
		5-11	≤1 time a month	≥2 times a month	≥2 times a week
		≥12	≤2 times a month	1-3 times a week	≥4 times a week
	Interference with normal activity	All	None	Some limitation	Extremely limited
	Rescue medication; use for symptoms	All	≤2 days/week	>2 days/week	Several times per day

**Table 4:** The thresholds for the classification of asthma control levels for both Digital Phenotype Score calculated using Partial Symptom Score and Digital Phenotype Score calculated using Total Symptom Score.

Asthma control level	Digital Phenotype Score (DPS)
Very poorly controlled	DPS≥1
Not well controlled	0.28≤DPS<1
Well controlled	DPS<0.28

### Data Availability

The study took comprehensive design- and technology-supported steps to ensure Health Insurance Portability and Accountability Act (HIPAA) compliance, privacy preservation, and data security. For example, each consented child was given a kHealth-ID by the nurse coordinator and retained so that child identifiable information did not leave the hospital clinic [21]. Throughout the data collection, only the deidentified data were made available for analysis by the kHealth system. A child’s real identity was not recorded, and any data exchange between DCH and other researchers ensured that the child’s identity information did not leave DCH. The data used for the study cannot be made publicly available because of HIPAA and other restrictions imposed by the approved IRB protocol. The dataset is accessible only to the clinician and the researchers involved in the study. Furthermore, it is available to the researchers only in anonymized form. All team members have completed relevant Collaborative Institutional Training Initiative program courses.

### Data Analysis

We performed a descriptive analysis of the data with IBM SPSS Statistics version 24 (IBM Corporation). We removed 13 children from our analysis who had data for less than 7 days to follow the NHLBI guideline, which requires a minimum of a week’s data to determine the child’s asthma control level [20]. This study presents an analysis of 82 children reporting data for more than 7 days. ACT scores were available from the EMRs for 57 of these children and were used for validation of the asthma control levels, calculated using the DPSs. We used Kendall Tau correlation metric to analyze the concordance between the asthma control level labeled using ACT scores and DPSs.

## Results

The average compliance of the kHealth kit was 75% (range: 9-100, SD=23). Of the children in our study cohort, 66% (54/82) were found to be highly compliant, 31% (25/82) were well compliant, and 4% (3/82) were found to be poorly compliant toward taking the kHealth app reading. The highly compliant and well compliant are defined using the kHealth app and taking a reading for at least 70% and 50%, respectively, of the study period. We classified the 82 children involved in our study into their asthma control level using the DPS-P and DPS-T. Using DPS-P, 30.5% of the children were classified as very poorly controlled, 26.8% as not well controlled, and 42.7% as well controlled. DPS-P classification (using scoring and threshold) adapts the ACT approach in the context of kHealth app. On the basis of DPS-T, 37% of the children were classified as very poorly controlled, 26% as not well controlled, and 38% as well controlled. Effectively, DPS-T classified a higher number of children as very poorly controlled or not well controlled as compared with DPS-P.

We used ACT scores recorded in the EMRs to understand how it corresponds to our classification based on the 2 DPS scales. ACT scores were available for 57 children of the 82 children who qualified for detailed evaluation. Tables 5 and 6 provide classification details using DPS-T and DPS-P based on adapted thresholds.

**Table 5:** The relationship between Asthma Control Test and the 2 versions of the Digital Phenotype Scores (N=57).

Asthma control labels	ACT^a^ score, n (%)	DPS-P^b^, n (%)	DPS-T^c^, n (%)
Very poorly controlled	9 (16)	20 (35)	23 (40)
Not well controlled	17 (30)	15 (26)	16 (28)
Well controlled	31 (54)	22 (39)	18 (32)

^a^ACT: Asthma Control Test.

^b^DPS-P: Digital Phenotype Score calculated using Partial Symptom Score.

^c^DPS-T: Digital Phenotype Score calculated using Total Symptom Score.

**Table 6:** Asthma Control Test and Digital Phenotype–based classification (N=57) where VPC is very poorly controlled asthma, NWC is not well controlled asthma, and WC is well controlled asthma. .

Asthma control labels	Children classified based on ACT^a^ score, n	Children classified based on DPS-P,^b^ n	Children classified based on DPS-T,^c^ n
Very poorly controlled (VPC)	9	VPC=7	VPC=8
		NWC=1	NWC=0
		WC=1	WC=1
Not well controlled (NWC)	17	VPC=11	VPC=13
		NWC=4	NWC=3
		WC=2	WC=1
Well controlled (WC)	31	VPC=2	VPC=2
		NWC=10	NWC=12
		WC=19	WC=17

^a^ACT: Asthma Control Test.

^b^DPS-P: Digital Phenotype Score calculated using Partial Symptom Score.

^c^DPS-T: Digital Phenotype Score calculated using Total Symptom Score.

As seen in Table 7, the DPS-P scale correctly classifies 7 out of 9 children as very poorly controlled. There was only 1 child with an ACT score of 11 (which is very poorly controlled) who was classified as well controlled according to DPS-P (=0.19) and not well controlled according to DPS-T (=0.39). We observed 2 children with the same DPS-P score of 1.33 (very poorly controlled), but they had 2 different ACT scores of 15 and 19, respectively, but both imply not well controlled. Although their DPS-P was the same, their DPS-T are 1.43 and 2.32, respectively, showing that the second child’s asthma condition is worse than the first one. We observed 2 children with DPS-P of 0.74 and 0.72, respectively, both not well controlled according to DPS-P criteria. According to their ACT, the same 2 children were classified as very poorly controlled (ACT score=13) and well controlled (ACT score=24). Furthermore, their DPS-T was 1.70 and 0.76, respectively. The latter’s scores are consistent with the DPS-P but also show that the first child is relatively worse off than the second child. Note also that differently classified children in the 3 categories were deployed in the allergy season, accounting for a higher SS during the allergy season, which was not captured by the ACT. Thus, DPS has the potential to provide more contemporary and timely gauge of a child’s asthma control.

Next, we provide further observations based on DPS-T and CCS. We observed that 50% of very poorly controlled children were poorly compliant, 20% well compliant, and 30% highly compliant. In the not well-controlled children cohort, 42.9% were poorly compliant, 28.6% well compliant, and 28.6% highly compliant. The well controlled children were 48.4% poorly compliant, 16.1% well compliant, and 35.5% highly compliant.

**Figure 5:**
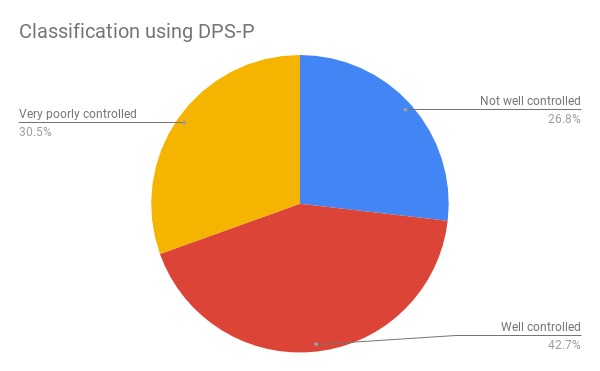
Distribution of Asthma Control Test score and Digital Phenotype Score calculated using Partial Symptom Score across the study population.

**Figure 6:**
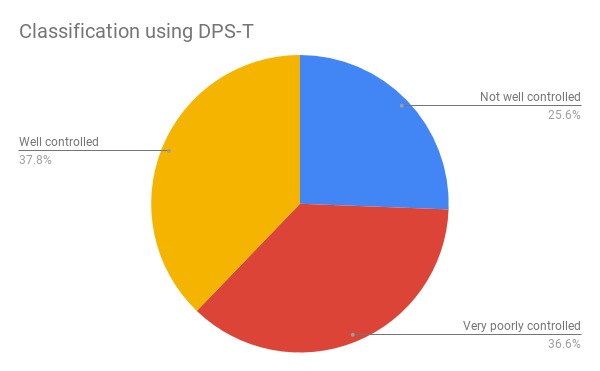
Distribution of Asthma Control Test Score and Digital Phenotype Score calculated using Total Symptom Score across the study population.

Figure 5 shows the distribution of ACT score and DPS-P. Figure 6 shows the distribution of ACT score and DPS-T.

To better quantify the relationship between the distribution of ACT scores and the DPS, we used Kendall Tau correlation metric to analyze the concordance between them [22,23]. We observed a negative correlation between ACT scores with both DPS-T and DPS-P and calculated them as −0.509** and −0.509**, respectively, (*P*<.01). The negative correlation accords well with that the fact the ACT scores and DPSs are inversely related. Unsurprisingly, we also observed a positive correlation between DPS-T and DPS-P as 0.921** (*P*<.01).

We also explored potential reasons for the varying asthma control by analyzing CCS, reflecting how well a child follows the asthma management protocol in terms of controller medication intake. We observed that 47.6% of the study participants were poorly compliant (according to CCS), 20.7% were well compliant, and 31.7% were highly compliant toward their controller medication use.

## Discussion

### Principal Findings

Suboptimal adherence to asthma therapy in children has been shown to result in poor disease control and increased hospitalization. Our study highlights this important issue, demonstrating a 50% compliance with controller medication in a cohort of children under the care of asthma specialists, which can be improved. In addition, almost 66% compliance with kHealth kit was achieved in this cohort. The 66% compliance with kHealth kit in this cohort appears to be highly encouraging, especially given the relative complexity of the kit (eg, need to connect with Wi-Fi or use a dongle) and the fact that DCH generally serves families in the medium to lower socioeconomic measures, including inner city. The kit compliance is the measure of the number of days the child took the kHealth kit reading during the deployment duration (1 or 3 months). This shows the potential to improve adherence by motivating children and parents. Several studies have demonstrated an increased adherence when data from electronic monitoring were shared with participants [9]. In children with asthma, an improvement in the use of preventive medication has been demonstrated when feedback was provided at each clinic visit [24]. The clinicians and researchers can be involved in patient-led and patient-centered digital health design [25]. It can encourage patients to share their data with their clinician or the researchers involved. It can be used to learn about the patient’s online networks and educate the patient by connecting with them and strengthening the network. The kHealth study offers an intervention for improving asthma control level as determined using the DPS. It demonstrates an opportunity for timely identification of children who are not well controlled or poorly controlled, and on the basis of their compliance, offer guidance for further intervention. For instance, for poorly compliant children, the parent or guardian can provide timely reminders to improve compliance for taking controller medication. In fact, this is also an opportunity to automatically generate friendly and timely reminders in a later version of our kHealth app, IRB approval permitting. For well complaint children, the clinician may decide to reevaluate medication and its dosage.

Several reasons can account for the discrepancy between assessments based on the ACT score and the DPS-P/DPS-T. The primary reason seems to be that the ACT was administered before the deployment of the kHealth kit and, in a number of cases, at the onset of the allergy season. Hence, there can be a mismatch between the relatively good asthma control level during trigger-free environment as compared with the asthma control level during allergy season. Furthermore, these same children are unlikely to return for a follow-up appointment (because their asthma is well controlled according to ACT and as dictated by the current practice are not required to take ACT at the end of the study period), and hence, we have no way to obtain ACT scores specifically summarizing the child’s condition during the allergy period for these specific study data. In addition, the ACT scores are based on child’s self-reporting and memory of episodes of past 4 weeks, running the risk of missing out on the fine-grained details that the kHealth can capture reliably and effortlessly (with its passive components). The kHealth app asks questions similar to ACT twice a day, providing a detailed day-to-day insight into the child’s condition. ACT scores provide an abstract, subjective, and cumulative picture of the child, whereas the kHealth app additionally provides an objective and a more granular insight into the child’s environment, activities, medication intake, and so on. Also note that ACT questions abstract away occurrences of a number of symptoms per day by considering only the number of days the symptoms appear, whereas DPS-T captures such additional fine-grained detail. The DPS-P was developed to approximate and generalize information sought in ACT questionnaire. Thus, DPS-T is able to distinguish between 2 children who experience a different number of symptoms every day that DPS-P is not sensitive to. As with ACT, we still do not distinguish the acuteness of different symptoms with respect to asthma. Using the DPS-based control levels and CCS, we can provide actionable insights (interventional steps) devised in consultation with our clinician partner as described in Table 7.

### Limitations

Children were enrolled on a first-come-first-serve basis, as the study was designed to demonstrate (1) feasibility of the kHealth system approach involving acceptability and usability, (2) robustness of the kHealth kit, quality, and consistency of measurements that provide clinically relevant data not currently available to the clinician (including assessing the relevance of patient-generated data and environmental data), and (3) maximize recruitment. Specifically, we had not planned the kHealth kit deployment for a child to coincide with their asthma exacerbation season or only in allergy season. We also did not deploy the kit for a longer duration (ie, half a year or year round) using the current IRB-approved protocol so as to straddle both nonallergy and allergy seasons to contrast the behaviors on normal days from allergy exposure days.

Several future options can further increase compliance, such as (1) use of more integrated sensors if the cost is acceptable [26], (2) higher monetary incentive for participation, (3) nonmonetary incentive including the clinician’s feedback during a follow-up consult, and (4) more close-up monitoring such as nurse coordinators calling the parent of the children who are delinquent in using the app during the early days of the participation.

**Table 7:** Actionable insight using Digital Phenotype Score and Controller Compliance Score.

Controller compliance	Very poorly controlled	Not well controlled	Well controlled
Highly compliant	Increase the medication dosage or change/add medication. Identify environmental triggers for mitigation.	Increase the medication dosage or change/add medication. Identify environmental triggers for mitigation.	Maintain therapy. Consider changing the medication or its dosage.
Well compliant	Increase the medication dosage or add medications; provide appropriate preventative suggestions.	Increase the medication dosage or add medications; provide appropriate preventative suggestions.	Maintain therapy. Consider changing the medication or its dosage.
Poorly compliant	Identify barriers to adherence and intervene.	Identify barriers to adherence and intervene.	Reassess diagnosis and modify therapy.

Children were given ACT before kHealth kit deployment, but the ACT was not repeated after the kHealth study period. Moreover, 57 of the 82 cohorts had recorded and available ACT scores. These factors made it difficult to more comprehensively validate our diagnosis based on the digital phenotype approach (DPS-P and DPS-T) against the ACT. Our extensive use of sensors and technology to collect a large variety of data, although novel, had resulted in initial challenges for reliable collection of data, ease of deployment, and use with implications on child compliance. To address these issues, the app underwent multiple design iterations to improve the data retention, usability in terms of intended functionality and connectivity, compliance, and reliability on passive sensing. In particular, 1 sensor used for nitric oxide measurement during the initial trial was removed by the manufacturer from the market and was replaced by the peak flow meter. The accuracy and usefulness of a number of sensors were assessed before starting the study [11].

### Conclusions

We have shown that the kHealth app is robust and reliable for use by the pediatric patient and can provide meaningful information to a clinician. We were able to clearly determine, at the cohort level, the relative distribution of children with different ACT scores into different control-level classes, as classified by digital phenotype approach. We also have evidence that tracking the children can not only shed light on their health condition but also provide actionable insight based on their adherence to asthma care plan. Specifically, we expect the digital phenotype and compliance data to be able to provide clinicians with detailed evidence much more transparently and in a more timely manner than the ACT. kHeath asthma was an observational study and lacked an intervention arm. Future trials assessing the impact of feedback derived from digital monitoring on adherence with asthma therapy in children are needed.

### Future Work

We plan to conduct a follow-up study that evaluates children over longer periods, covering both allergy and nonallergy seasons. We would like to identify factors affecting asthma at a personalized level as a means to detect the triggers that cause the asthma-related symptoms, monitor the control level, and predict the potential for asthma exacerbation to minimize emergency room visits. This can also help us educate parents and children to improve asthma management and to empower clinicians via an evidence-based approach to obtain better outcomes for each child. We expect the digital phenotype approach and the monitoring of controller medication compliance developed here to be useful in modifying the care protocol and will use them to design a follow-up study as an interventional step. At a broader level, we seek to enhance health care and chronic disease management by aggregating, analyzing, and personalizing the use of relevant physical, cyber, and social data obtained from wearables, sensors, mobile apps, EMRs, Web-based information, and social media. We also seek to develop tailored health management strategies suitable for all ages and at different levels, including self-monitoring, self-appraisal, self-management, intervention, prediction, and tracking disease progression, as identified in our Augmented Personalized Healthcare approach [27,28]. Ultimately, we expect evidence-based disease management to help in reducing the overall cost of care while simultaneously improving quality of life.

## Appendix

Multimedia Appendix 1 kHealth Wikipedia and kHealthDash Demo Video.

## Abbreviations

ACT: Asthma Control Test
AcS: Activity Score
AwS: Awakening Score
CCS: Controller Compliance Score
DCH: Dayton Children’s Hospital
DPS: Digital Phenotype Score
DPS-T: Digital Phenotype Score calculated using Total Symptom Score
DPS-P: Digital Phenotype Score calculated using Partial Symptom Score
EMR: electronic medical record
HIPAA: Health Insurance Portability and Accountability Act
IRB: institutional review board
NHLBI: National Heart Lung Blood Institute
PSS: Partial Symptom Score
RS: Rescue Score
SS: symptom score
TSS: Total Symptom Score
