# Supplementary material for: “How Is My Child’s Asthma?” Digital Phenotype and Actionable Insights for Pediatric Asthma
Source: JMIR Pediatr Parent. 2018 Nov 30;1(2):e11988. doi: 10.2196/11988 (PMC6469868; doi:10.2196/11988)
Supplement: Multimedia Appendix 1 [file pediatrics_v1i2e11988_app1.pdf]

MULTIMEDIA APPENDIX

kHealth Wikipedia-
http://wiki.knoesis.org/index.php/KHealth:_Semantic_Multisensory_Mobile_Approach_to_Personalized_Asthma_Care#Publications

kHealthDash Demo video- https://www.youtube.com/watch?v=yUgXCPwc55M
